# Habitual cigarette smoking attenuates shear‐mediated dilation in the brachial artery but not in the carotid artery in young adults

**DOI:** 10.14814/phy2.14369

**Published:** 2020-02-15

**Authors:** Kazuya Suzuki, Takuro Washio, Shingo Tsukamoto, Kazunori Kato, Erika Iwamoto, Shigehiko Ogoh

**Affiliations:** ^1^ Department of Biomedical Engineering Toyo University Kawagoe‐Shi Saitama Japan; ^2^ Research Fellow of Japan Society for the Promotion of Science Tokyo Japan; ^3^ School of Health Sciences Sapporo Medical University Sapporo Japan

**Keywords:** endothelial function, internal carotid artery, nitric oxide, reactive oxygen species, shear rate

## Abstract

In the present study, we hypothesized that habitual cigarette smoking attenuates endothelial function in the cerebral circulation as well as that of the peripheral circulation in young adults. To test this hypothesis, we measured cerebrovascular and peripheral flow‐mediated dilation (FMD) in young smokers and nonsmokers in the present study. Ten healthy nonsmokers and 10 smokers participated in the study. We measured blood velocity and diameter in the brachial artery and internal carotid artery (ICA) using Doppler ultrasound. We identified shear‐mediated dilation in the brachial artery and ICA by the percentage change in peak diameter during hyperemia stimulation (reactive hyperemia and hypercapnia). We measured the baseline diameter and the shear rate area under the curve from the onset of hyperemia to peak dilation in the brachial artery and ICA, finding the measurements of the smokers and those of the nonsmokers did not differ (*p* > .05). In contrast to brachial FMD (5.07 ± 1.79% vs. 7.92 ± 3.01%; smokers vs. nonsmokers, *p* = .019), FMD in the ICA was not attenuated in the smokers compared with that of the nonsmokers (5.46 ± 2.32% vs. 4.57 ± 2.70%; *p* = .442). These findings indicate that in young healthy smokers, cerebral endothelial function was preserved, and the response of cerebral endothelial function to smoking was different from that of peripheral vasculature.

## Graphical abstract

Peripheral FMD was lower in young healthy smokers than that in nonsmokers, indicating that habitual smoking attenuates peripheral endothelial function. In contrast, cerebral endothelial function was preserved in young healthy smokers. These findings suggest that the response of cerebral endothelial function to smoking is different from that of peripheral vasculature.

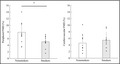

## 1 INTRODUCTION

Habitual cigarette smoking is well known to cause hypertension (Virdis, Giannarelli, Neves, Taddei, & Ghiadoni, 2010), lung damage (Vial, 1986), dysfunction of peripheral vascular function (Ambrose & Barua, 2004), and subsequent impairment of systemic vascular function. It has been reported that habitual cigarette smoking is associated with not only coronary and peripheral vascular diseases but also the development of cerebrovascular disease, including stroke (Shah & Cole, 2010), cognitive decline (Anstey, Sanden, Salim, & O'Kearney, 2007), and Alzheimer's disease (Brenner et al., 1993). These epidemiologic findings suggest that cigarette smoking—by affecting cerebral vasculature—increases the risk of cerebrovascular disease. Most previous studies regarding cigarette smoking, however, have focused on peripheral vascular function, which is different from that of cerebral vasculature. Using a transcranial Doppler ultrasound, some previous studies (Cruickshank, Neil‐Dwyer, Dorrance, Hayes, & Patel, 1989; Terborg, Birkner, Schack, & Witte, 2002; Terborg, Bramer, Weiller, & Rother, 2002) have examined the immediate effect of smoking a cigarette on cerebral hemodynamics, and demonstrated that smoking increases blood flow velocity in the middle cerebral artery (MCA). In addition, other studies (Silvestrini, Troisi, Matteis, Cupini, & Bernardi, 1996; Terborg, Birkner, et al., 2002) reported that cerebrovascular reactivity to hypercapnia was reduced after cigarette smoking. These findings suggest that this acute negative effect of smoking a cigarette on cerebral hemodynamics could contribute partly to the increased risk of cerebral infarction in current smokers. However, these studies investigated the immediate effect of smoking a cigarette on cerebral circulation. Moreover, it has been suggested that the transcranial Doppler data they used may be reflected by smoking‐induced vasoconstriction of the isolated vessel (Terborg, Bramer, et al., 2002). Therefore, both the effects of habitual cigarette smoking on cerebral circulation and the physiological mechanism triggering the onset of cerebrovascular diseases in smokers remain unclear.

Vascular function is determined by many physiological factors. Among these, endothelial function plays several critical roles in the maintenance of vascular homeostasis—including modulation of vascular tone, maintenance of blood circulation, regulation of inflammation and immune response, and angiogenesis (Deanfield, Halcox, & Rabelink, 2007). Endothelial dysfunction is caused by a decrease in the production of nitric oxide (NO) from the endothelium and abnormal secretion of other vasoactive substances that attenuate vasodilatory response and cause vasoconstriction. Moreover, endothelial dysfunction has been demonstrated to be associated with a risk of developing various cardiovascular and cerebrovascular diseases such as arteriosclerosis (Deanfield et al., 2007; Targonski et al., 2003).

Flow‐mediated dilation (FMD), for instance in response to reactive hyperemia, is widely used as a noninvasive method for evaluating peripheral endothelial function. A previous study regarding smoking reported that peripheral FMD was lower in young smokers than it was in nonsmokers, indicating that habitual smoking attenuates peripheral endothelial function (Ozaki, Hori, Ishibashi, Nishio, & Aizawa, 2010). It has therefore been considered that this attenuation in peripheral FMD contributes to the development and progression of atherosclerotic cardiovascular disease (Ras, Streppel, Draijer, & Zock, 2013). Under this background, we hypothesized that habitual cigarette smoking attenuates endothelial function in the cerebral circulation as well as that of the peripheral circulation in young humans. Recent elegant studies (Carter et al., 2016; Hoiland et al., 2017) have proved that shear rate (SR) contributes to the dilation in the internal carotid artery (ICA) during hypercapnia, and this shear‐mediated dilation of ICA occurs independently of sustained in hypercapnia. Therefore, as with the peripheral conduit arteries, SR plays a key role in carotid artery vasodilatation (Hoiland et al., 2017; Smith et al., 2019). The hypercapnia‐induced shear‐mediated dilation in the ICA as a cerebrovascular FMD has been used for the characterization of cerebrovascular endothelial function in previous studies (Carter et al., 2016; Hoiland et al., 2017; Iwamoto, Bock, & Casey, 2018a, b; Smith et al., 2019). In addition to measure peripheral FMD, here, to test this hypothesis, we assessed the cerebrovascular FMD using this new technique in young smokers and nonsmokers.

## 2 METHODS

### 2.1 Subjects

The institutional review board of Toyo University approved all procedures of the present study, which conformed to the ethical principles of the Declaration of Helsinki (IRB TU2017‐022). Twenty healthy young people—10 nonsmokers (10 men, 21 ± 1 years) and 10 smokers (nine men and one woman, 21 ± 1 years)—participated in the present study (Table 1). A power test was performed to determine the required number of subjects in the present study using brachial FMD data between smoker and nonsmoker in three subjects of the prestudy. For the power calculation an unpaired *t*‐test (α level = 0.05; power = 0.95) indicated that the sample size should be *n* > 9 to obtain a significant difference in each group. Hence, we decided to measure 10 subjects in each group. We calculated the extent of cigarette exposure, referred to as “pack‐years,” using the following equation: pack‐years = (number of cigarettes per day) × (number of years of smoking)/20 (Amato et al., 2013). We instructed the participants to abstain from a light meal for 2 hr prior to the study and to refrain from strenuous exercise, alcohol, and caffeine for 24 hr prior to the study. We instructed the participants in the smoker group to refrain from smoking for at least 12 hr before arrival in the laboratory (Vafaee et al., 2015). Acute smoking cessation may cause not only physical changes but also metal stress. In the present study, there is no different in hemodynamics between groups. In addition, previous study (D'Urzo et al., 2019) demonstrated that mental stress does not affect the FMD. Thus, we believe that effect of acute smoking cessation may be minimal in the present study.

**Table 1:** Participant characteristics

	Nonsmokers	Smokers	*P* values
Sex (male/female)	10/0	9/1	NA
Age (years)	21 ± 1	21 ± 1	.331
Height (cm)	173 ± 4	171 ± 8	.433
Weight (kg)	65 ± 13	63 ± 12	.768
BMI (kg/m^2^)	22 ± 4	22 ± 3	.997
HR (bpm)	61 ± 7	59 ± 5	.590
SV (ml)	101 ± 20	87 ± 19	.126
MAP (mmHg)	85 ± 8	80 ± 9	.155
P_ET_CO_2_ (mmHg)	41 ± 3	40 ± 2	.178
Pack‐years	NA	1.4 ± 1.5	NA

Note

Values are expressed as mean ± *SD*.

Abbreviations: BMI, body mass index; HR, heart rate; MAP, mean arterial pressure; P_ET_CO_2,_ end‐tidal partial pressure of carbon dioxide; SV, stroke volume; Pack‐years = (number of cigarettes per day) × (number of years of smoking)/20.

### 2.2 Experimental procedure

When the participants arrived at the laboratory, they rested on beds in a supine position for at least 15 min before the measurements commenced. After this resting period, we performed the following measurements while they remained in a supine position in a room at a constant temperature (23°C–24°C).

### 2.3 Hemodynamic measurements

We continuously measured heart rate (HR) using a lead II electrocardiogram (bedside monitor, BMS‐2401; Nihon Kohden, Japan) and beat‐to‐beat arterial blood pressure using finger photoplethysmography (Finapres Medical Systems BV, Netherlands). We determined stroke volume (SV) from the blood pressure waveform using the Modelflow software program, which incorporates the sex, age, height, and weight of the subject (Beat Scope1.1; Finapres Medical Systems BV). We analyzed P_ET_CO_2_ from respiratory gas sampled via a leak‐free mask using a gas analyzer (AE‐310S; Minato Medical Science Co.).

### 2.4 Doppler measurements

Trained investigators measured arterial diameter and blood velocity in the brachial artery and ICA using Doppler ultrasound (Vivid i, GE Medical Systems, Tokyo, Japan) equipped with 13‐MHz linear array transducers. They obtained the diameter and blood velocity in the right brachial artery in the longitudinal section 3 cm to 5 cm above the antecubital fossa, and measured those in the ICA 1.0 cm to 1.5 cm cranial to the carotid bifurcation from the right side of the neck with a Doppler beam angle of 60 degrees. We obtained both the brachial artery and the ICA diameters using high resolution ultrasound in B‐mode images. We captured arterial images and associated velocity waveforms at 30 Hz using a capture box (The Epiphan Capture Tool, Epiphan Systems Inc.) and stored them in a computer to identify peripheral and cerebrovascular FMD. All Doppler measurements were performed by the same operator.

### 2.5 Peripheral FMD measurement

We measured blood velocity and diameter in the right brachial artery using Doppler ultrasound (Vivid i, GE Medical Systems). We identified peripheral FMD by the percentage change in peak diameter during ischemia‐induced reactive hyperemia stimulation from the baseline value. To examine the peripheral FMD response, we used an inflation/deflation pneumatic cuff to provide the ischemic stimulus (Harris, Nishiyama, Wray, & Richardson, 2010). We recorded baseline scans assessing resting vessel diameter and velocity over 2 min. We then inflated the cuff to >250 mmHg for 5 min. We resumed diameter and blood velocity recordings 30 s before cuff deflation and continued recording for 2 min thereafter (Corretti et al., 2002).

### 2.6 Cerebrovascular FMD measurement

We measured blood velocity and diameter in the right ICA using Doppler ultrasound (Vivid i, GE Medical Systems). We identified shear‐mediated dilation in the ICA by the percentage change in peak ICA diameter during hypercapnia stimulation induced using high carbon dioxide (CO_2_) gas from the baseline diameter (Carter et al., 2016). We ran trials consisting of a 2 min baseline period followed by 3 min of hypercapnia. The hypercapnia stimulation comprised inspiration of high carbon dioxide concentration gas (target end‐tidal partial pressure of carbon dioxide (P_ET_CO_2_); +10 mmHg from individual baseline value) from a gas mask attached to a low resistance two‐way valve with a flowmeter (AE‐310S; Minato Medical Science Co.). By injecting pure CO_2_ using a mixing chamber (250 ml gas blender; Arco system), we ensured that all participants breathed a mixture of room air and CO_2_. The subjects were instructed to adjust their resting respiratory rate using a metronome during the measurement.

### 2.7 Data analysis

We analyzed all data except FMD data offline with signal processing software (Power Lab 16/s, ADInstruments). We averaged continuously measured hemodynamic variables of the initial 2 min (baseline) and of the 10 s before peak ICA diameter during the hypercapnia condition. We then analyzed the diameter and mean blood velocity (V_mean_) during FMD tests at 30 Hz using custom‐designed edge‐detecting and wall‐tracking software (version 2.0.1, S‐13037, Takei Kiki Kogyo) (Iwamoto, Bock, & Casey, 2018b). We detected brachial artery data using an algorithm reported previously (Black, Cable, Thijssen, & Green, 2008). We interpolated ICA data to 1 Hz and filtered them using the two‐stage filtering process (median filter and Savitzky‒Golay finite impulse response smoothing filter) (Iwamoto et al., 2018b) with custom software (Python version 3.6.1, Python Software Foundation). We calculated SR in the brachial artery and ICA by the equation; 4 × V_mean_/ arterial diameter. We defined the baseline diameter and shear rate (*D*_base_, SR_base_) in the brachial artery as the mean value measured during baseline and defined those values in the ICA as the median value during baseline. We determined the peak diameter and SR (*D*_peak_, SR_peak_) in the brachial artery and ICA as a peak value from the onset response curve to hyperemia to *D*_peak_. We calculated shear‐mediated dilation and the percent change in SR using peak and baseline values [(*D*_peak_ – *D*_base_)/*D*_base_ × 100]. We quantified the SR area under the curve (SR_AUC_) as the area from the time of deflation and hypercapnia to the time of D_peak_ and calculated it using the trapezoidal rule; ∑ [1/2 (*x*i + 1 − *x*i) (*y*i + 1 − *y*i) + (*x*i + 1 − *x*i) (*y*i − *z*)], where *x* represents time, *y* represents SR, and *z* represents SR_base_ (Iwamoto et al., 2018a). *D*_base_, *D*_peak_, SR_base_, and SR_peak_ were automatically detected or calculated by the customized software (Python).

### 2.8 Statistical analysis

We express all data as mean ± standard deviation (*SD*). We analyzed data via the unpaired student's *t* test (nonsmokers and smokers) and P_ET_CO_2_ data via two**‐**way analysis of variance (ANOVA) [group (nonsmokers vs. smokers) × time (baseline vs. hypercapnia)] followed by a Bonferroni post hoc test (SPSS version 25; IBM). We have ensured normal distribution by used Shapiro‒wilk test in our data. Each peripheral FMD (*p* = .404, and *p* = .458; smokers and nonsmokers), cerebrovascular FMD (*p* = .386, and *p* = .273) were normal distributed. We considered *p* values < 0.05 to indicate statistical significance.

## 3 RESULTS

**Figure 1:**
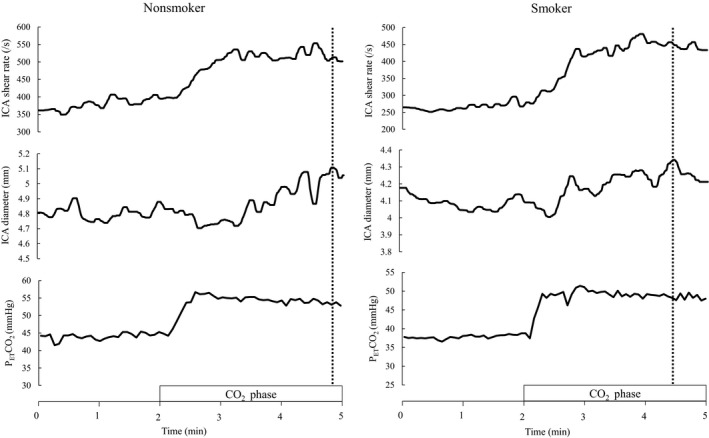
Representative individual response of diameter and shear rate in the internal carotid artery (ICA) during cerebrovascular hyperemia protocol in nonsmokers and smokers; 2 min of baseline followed by 3 min of hypercapnia (+10 mmHg from the baseline end‐tidal partial pressure of carbon dioxide [P_ET_CO_2_]). Vertical broken line denotes a peak vasodilation of ICA

**Figure 2:**
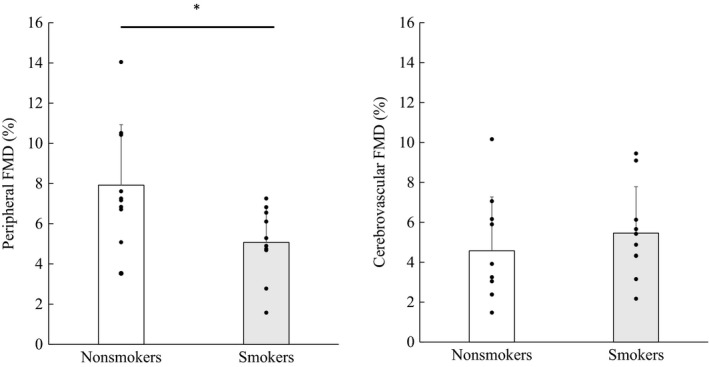
Peripheral (brachial artery) and cerebrovascular (internal carotid artery) flow‐mediated dilation (FMD). Values are mean ± *SD* (*n* = 10). *P* value represents unpaired student's *t* test results. The bar and closed circles represent average and individual value. *p = .019 versus nonsmokers

There was no significant difference in the subject's characteristics between groups (*p* > .05 for all, Table 1). In Figure 1 we present representative individual time course response of SR and diameter in the ICA and P_ET_CO_2_ to hyperemia (hypercapnic stimulation). There were no differences in hypercapnia‐induced increases in P_ET_CO_2_ at the peak of dilation between group and time (baseline to hypercapnia: nonsmokers, 41 ± 3 mmHg to 51 ± 3 mmHg, smokers, 40 ± 2 mmHg to 48 ± 3 mmHg; interaction effect, *p* = .379). All parameters for the FMD in the ICA measured in the smokers did not differ significantly from those of nonsmokers; *D*_base_, *D*_peak_, SR_base_, SR_peak_, SR_AUC_, and peak time (*p* > .05 for all, Table 2). Also, the FMD in the ICA in the smokers was not attenuated compared with that in the nonsmokers (5.46 ± 2.32% vs. 4.57 ± 2.70%; *p* = .442, Figure 2).

**Table 2:** Flow‐mediated dilation in the brachial artery and internal carotid artery profile

	Nonsmokers	Smokers	*P* values
Brachial artery			
D_base_ (mm)	3.72 ± 0.45	3.55 ± 0.54	.446
D_peak_ (mm)	4.01 ± 0.45	3.73 ± 0.60	.252
SR_AUC_ (a.u.)	21,705 ± 9,330	20,442 ± 6,432	.729
Peak time (s)	56 ± 24	52 ± 31	.738
Internal carotid artery			
D_base_ (mm)	4.81 ± 0.54	4.54 ± 0.49	.264
D_peak_ (mm)	5.02 ± 0.55	4.79 ± 0.54	.347
SR_base_ (/s)	274 ± 69	286 ± 30	.627
SR_peak_ (/s)	401 ± 88	412 ± 53	.736
SR_AUC_ (a.u.)	50,179 ± 16,508	57,415 ± 13,304	.295
Peak time (s)	138 ± 31	159 ± 34	.173

Note

Values are mean ± *SD*. *D*_base_, baseline diameter; *D*_peak_, peak diameter; SR_base_, baseline shear rate; SR_peak_, peak shear rate from the onset of hyperemia to the peak dilation; SR_AUC_, shear rate area under the curve from the onset of hyperemia to peak dilation; Peak time, time to peak dilation from the onset of hyperemia.

For all parameters of the peripheral FMD measured between nonsmokers and smokers—*D*_base_, *D*_peak,_ SR_AUC_, and Peak time—none differed significantly (*p* > .05 for all, Table 2). In contrast to results for the FMD in the ICA, the brachial FMD in the smokers was significantly smaller than that in the nonsmokers (5.07 ± 1.79% vs. 7.92 ± 3.01%; *p* = .019, Figure 2). In Figure 2, there was one subject who showed a low value in smoker, but 14% nonsmokers were included. The peripheral FMD in smoker was significantly lower than that in all nonsmokers (*p* = .019) or nonsmokers without one subject who has a low value (*p* = .039).

## 4 DISCUSSION

The present study for the first time examined the effects of smoking on cerebral endothelial function. As in previous studies, peripheral (brachial) FMD was attenuated in smokers compared with nonsmokers. However, in contrast to our hypothesis, cerebrovascular (ICA) FMD in smokers was not different from that of nonsmokers. These findings indicate that cerebral endothelial function was preserved in young healthy smokers, and that the response of cerebral endothelial function to smoking was different from that of peripheral vasculature.

In the present study, peripheral FMD was significantly lower in smokers than that in nonsmokers (Figure 2), indicating that peripheral endothelial function was attenuated in young healthy smokers. This finding is consistent with previous studies demonstrating that habitual cigarette smoking in young adults impairs peripheral endothelium‐dependent vasodilation (Barua et al., 2001; Celermajer et al., 1993; Yufu, Takahashi, Hara, Saikawa, & Yoshimatsu, 2007). Endothelial dysfunction is well known as one of the earliest pathophysiological effects of various risk factors for atherosclerosis (Celermajer, Sorensen, Bull, Robinson, & Deanfield, 1994; Feletou & Vanhoutte, 2006). These findings suggest that cigarette smoking‐induced elevation in the risk of cardiovascular disease may be associated with endothelial dysfunction. Additionally, previous studies have demonstrated that decreases in nitric oxide (NO) bioavailability and increases in the generation of reactive oxygen species (ROS) play critical roles in endothelial dysfunction in smokers (Barua et al., 2001; Fennessy, Moneley, Wang, Kelly, & Bouchier‐Hayes, 2003; Raij, DeMaster, & Jaimes, 2001). Fratta Pasini et al. (2012) reported a further novel consequence of increased ROS in young smokers with endothelial dysfunction as the repression of nuclear erythroid‐related factor 2/related antioxidant genes pathway, leading to glutathione depletion. Although an index of oxidative stress was not measured in the present study, these findings provide the possibility that cigarette smoking causes endothelial dysfunction in peripheral vasculature via an increase in oxidative stress, and a consequent increase in the risk of cardiovascular disease.

An increase in oxidative stress has been reported to adversely affect not only peripheral vasculature but also the regulation of cerebral blood flow (Grochowski, Litak, Kamieniak, & Maciejewski, 2018). An increase in ROS impairs cerebral autoregulation (Bailey et al., 2011), a response of cerebral vasculature to ROS apparently associated with several other pathways, including damaged endothelial cells (Faraci, 2006). It is therefore possible that an increase in ROS also impairs cerebral endothelial function. However, endothelial function has been demonstrated to be different between organs, and potentially also between different vascular beds within the same organ (Aird, 2007). Especially, the cerebral endothelium is one of the most specific types, probably since it is an important element of the well‐known blood–brain barrier (BBB) (Daneman & Prat, 2015). These findings indicate that endothelial function in the carotid artery is different from that of peripheral circulation, and therefore, the effect of cigarette smoking on endothelial function in cerebral circulation was expected to be different from that in peripheral circulation. Here, to examine this question, we evaluated cerebral endothelial function in smokers via hypercapnia‐induced shear‐mediated dilation in the ICA using techniques recently introduced by Carter et al. (2016). Contrary to our hypothesis, cerebrovascular FMD was not attenuated in young smokers (Figure 2). Cerebral endothelial cells are equipped with superoxide dismutase as well as an extensive defense system (glutathione, glutathione peroxidase, glutathione reductase, and catalase) against oxidative stress (Tayarani, Chaudiere, Lefauconnier, & Bourre, 1987; Vatassery, 1998). Indeed, reports of a regional heterogeneity in the oxidative stress response suggest that the contribution of cerebral circulation to free radical formation during oxidative stress conditions (such as hypoxia) was less than that of peripheral circulation (Bailey et al., 2018). These findings suggest that the protection of cerebral vasculature against oxidative stress may be greater than that of peripheral vasculature. Against this background, our finding regarding a difference in endothelial function between cerebral and peripheral circulation may be reasonable. However, this physiological mechanism of the different response of endothelial function between cerebral and peripheral circulation remains unknown.

Peripheral endothelial dysfunction has been demonstrated to be associated not only with peripheral arterial disease such as arteriosclerosis but also with the risk of developing cerebrovascular disease (Targonski et al., 2003). Therefore, the peripheral FMD test strongly predicts cardiac events (Green, Jones, Thijssen, Cable, & Atkinson, 2011), potentially allowing for preclinical detection of future development of cardiovascular disease (Celermajer et al., 1992). Similarly, cerebrovascular FMD may be a useful marker for assessing the risk of developing cerebrovascular disease. A different cerebrovascular FMD compared with peripheral FMD in smokers indicates that cigarette smoking increases the risk of cardiovascular disease more than it does the risk of cerebrovascular disease, and epidemiological studies support this concept (Banks et al., 2019; Ding et al., 2019). However, further investigations are necessary to identify the different effects of smoking cigarette on cardiovascular or cerebrovascular endothelial function and development of these disease risks.

There may be some limitations in this study. First, we did not measure a marker of ROS, as a correlation between FMD in brachial arteries, although an oxidative stress marker (malondialdehyde) has been reported previously (Kaya et al., 2012). Also, the other previous studies demonstrated that smoking‐induced decrease in FMD is due to increased oxidative stress (Tanriverdi et al., 2006). Therefore, it is also reasonable to consider that the decrease in peripheral FMD, in the present study, is also due to the increase in ROS in smokers. Second, we did not measure blood viscosity, which affects shear stress. It has been identified that smokers tend to have higher blood viscosity than that of nonsmokers (Shimada et al., 2011). Therefore, smoker may have a greater shear stress stimulus. Third, there was no inhibition of pharmacological endothelial NO synthase. To confirm endothelial dependence, it is necessary to determine the effect of elevated shear‐mediated vasodilation in the ICA before and after NO blockade. Forth, the subjects were asked to abstain food for 2 hr prior to this study. It may be not certainly enough to remove the acute effects of nutrient intake on endothelial function. However, the subjects were asked to have a light meal as the breakfast. Also, even previous studies (Esen et al., 2004) using the similar method regarding the breakfast observed a significant decrease the brachial FMD in smokers. Finally, changes in shear stress as a hyperemia stimulus during cerebrovascular FMD measurement were different from that of peripheral FMD; shear stress was increased sharply or slowly, respectively. To elucidate the sensitivity of peripheral and cerebral endothelium by shear stress, further study, such as matched SR_AUC_ and shortening the time of CO_2_ inhalation rapidly, as well as reactive hyperemia stimuli, is necessary (Hoiland et al., 2017).

In summary, in smokers, compared with nonsmokers, the peripheral endothelial function was attenuated while cerebrovascular endothelial function was unchanged in young adults. These findings suggest that cigarette smoking may increase the risk of peripheral vascular disease earlier than that of cerebral vascular disease. Importantly, the effect of cigarette smoking on cerebrovascular circulation was different from that of peripheral vasculature, and cerebral vascular function may be more protected from oxidative stress compared with peripheral circulation.

## CONFLICT OF INTEREST

No conflict of interest, financial or otherwise, is declared by the authors.

## AUTHOR CONTRIBUTIONS

Author contributions: K.S. and S.O. were involved in conception and design of research and drafted the manuscript. K.S., T.W., S.T., and S.O. performed experiments. K.S., S.T., and E.I. analyzed data and interpreted the results of experiments. K.S. prepared figures. All authors edited and revised manuscript and approved final version of manuscript.
